# The formation and evolution of flower coloration in *Brassica* crops

**DOI:** 10.3389/fgene.2024.1396875

**Published:** 2024-05-30

**Authors:** Xuewei Li, Mingmin Zheng, Qingqin Gan, Jiang Long, Haiyan Fan, Xiaoqing Wang, Zhilin Guan

**Affiliations:** ^1^ Jiangxi Provincial Institute of Traditional Chinese Medicine, Jiangxi Research Center for Protection and Development of Traditional Chinese Medicine Resources, Key Laboratory of Germplasm Selection and Breeding of Chinese Medicinal Materials, Nanchang, Jiangxi, China; ^2^ College of Life Sciences, Xinyang Normal University, Xinyang, China; ^3^ Wuhan Botanical Garden, Chinese Academy of Sciences, Wuhan, Hubei, China

**Keywords:** flower coloration, *Brassica* crops, carotenoids, flavonoids, formation, evolution

## Abstract

The flower coloration of *Brassica* crops possesses significant application and economic value, making it a research hotspot in the field of genetics and breeding. In recent years, great progress has been made in the research on color variation and creation of *Brassica* crops. However, the underlying molecular mechanisms and evolutional processes of flower colors are poorly understood. In this paper, we present a comprehensive overview of the mechanism of flower color formation in plants, emphasizing the molecular basis and regulation mechanism of flavonoids and carotenoids. By summarizing the recent advances on the genetic mechanism of flower color formation and regulation in *Brassica* crops, it is clearly found that carotenoids and anthocyanins are major pigments for flower color diversity of *Brassica* crops. Meantime, we also explore the relationship between the emergence of white flowers and the genetic evolution of *Brassica* chromosomes, and analyze the innovation and multiple utilization of *Brassica* crops with colorful flowers. This review aims to provide theoretical support for genetic improvements in flower color, enhancing the economic value and aesthetic appeal of *Brassica* crops.

## 1 Introduction

Flowers of angiosperms serve as reproductive organs, flower coloration is a crucial trait for the survival and reproduction of plants, which can not only attract insects to pollination, but also maintains the energy balance under different lighting conditions to protect flower organs (Clegg and Durbin, 2000; Ariizumi et al., 2014). As an apparent trait, flower color has been widely used as a marker or target trait across genetics, molecular biology, ecology, evolutionary biology and crop breeding (Liu et al., 2004). Additionally, given its significant ornamental value, creating novel and diverse flower colors has remained a primary objective in flower breeding (Li et al., 2023). Flower color is mainly attributed to the pigments present in petals, carotenoids and flavonoids are two main large classes of natural pigments contributing to most of colors in plants like yellow, orange and red (Jin et al., 2014; Ohmiya, 2013). In the last two decades, the corresponding biosynthetic pathways have been well characterized and elucidated, which provide a foundation for understanding the development and molecular basis of flower color (Koes et al., 2005; Ruiz-Sola and Rodriguez-Concepcion, 2012).

*Brassica* species are grown worldwide as important oil crops, vegetables and feed crops. There are mainly six representative cultivated *Brassica* crops including three diploid species, *B. rapa* (2n = 20, AA), *B. nigra* (2n = 16, BB), and *B. oleracea* (2n = 18, CC), and three allotetraploid species, *B. juncea* (2n = 36, AABB), *B. napus* (2n = 38, AACC), and *B. carinata* (2n = 34, BBCC). These three allotetraploids originate from interspecific hybridizations among the remaining three diploid species, resulting in a more complex genetic basis (Nagaharu, 1935). *Brassica* crops exhibit flower color diversity that more than 63 different colored rape varieties have been obtained in China (Xiao et al., 2021). However, to date the molecular mechanisms underlying flower color diversity of *Brassica* crops remain elusive. Early studies reported that anthocyanins are responsible for the red and purple coloration on leaves, stems, petals and floral meristem of *Brassica* crops, while the total content of carotenoids showed significant differences between yellow-flowered and white- flowered varieties (Chiu and Li, 2012; Zhang et al., 2015; Li et al., 2023). In recent years, with multi-omics approach, numerous flavonoids (including anthocyanins) and carotenoids have been isolated and characterized from different-colored petals of several *Brassica* crops (Li et al., 2015; Yin et al., 2019; Ye et al., 2022), and a number of structural and regulatory genes involved in the flavonoids and carotenoids biosynthetic pathways have been identified as flower color controlled genes (Chen et al., 2023; Guan et al., 2023). Thus, much have been learned about the mechanism of flavonoids and carotenoids on flower color formation in *Brassica* crops. This paper is expected to summarize the regulation mechanisms of flower color formation in *Brassica* crops, as well as elucidating the relationship between different phenotypic and genotypic evolutions, which are crucial for harnessing and cultivating diverse flower colors in *Brassica* species and beyond.

## 2 The formation mechanism of flower color in plants

The coloration of petals is determined mainly by the presence and relative abundances of three pigment classes including carotenoids, flavonoids, and betalains (Zhao et al., 2022). Carotenoids and flavonoids, in particular, govern the hues exhibited by a vast array of plants, while betalains exist specifically in Caryophyllales plants (except Caryophyllaceae and Molluginaceae), and chlorophyll in cells would affect the final color (Zhao and Tao, 2015). The diversity in petal coloration among different plants arises from the unique combinations and concentrations of these pigments. The biosynthetic pathways responsible for the formation of these pigments are intricately regulated by a range of structural and regulatory genes. Variations in the types, quantities, and sequence characteristics of these encoding genes, as well as differences in their expression patterns within cells, constitute the molecular basis for the observed chromatic aberrations in petals (Ohmiya, 2013). By understanding the genetic and biochemical mechanisms underlying petal coloration, it can gain insights into the remarkable diversity of plant phenotypes and their adaptation strategies in nature.

### 2.1 Carotenoids biosynthetic pathways and key genes

Carotenoids are a class of fat-soluble pigments widely distributed in flowers, fruits, leaves and roots, primarily control the yellow and orange to red colorations. These pigments are a group of 40-carbon triterpenes composed of eight isoprene basic units linked end-to-end. They are synthesized through the isoprene pathway and are typically localized in plastid or chromoplast (Zhao et al., 2022). The backbone of carotenoids consists of a main chain containing nine double bonds, with each end of the chain being cycled to form a β-violone ring. Carotenoids can be categorized into two groups, non-oxygenated carotenoids (Carotenes, such as α-carotene, β-carotene, etc.) and oxygenated carotenoids (Xanthophyll, such as zeaxanthin, lutein, antheraxanthin, etc.). Oxygenated carotenoids further encompass bicyclic oxides and monocyclic oxides (Grotewold, 2006; Schweiggert et al., 2012). Xanthophylls exhibiting light yellow color, which include lutein, zeaxanthin, antheraxanthin, violaxanthin, and neoxanthin, are the main carotenoids imparting yellow colorations of petals and flesh (Jin et al., 2014; Niaz et al., 2023).

**FIGURE 1:**
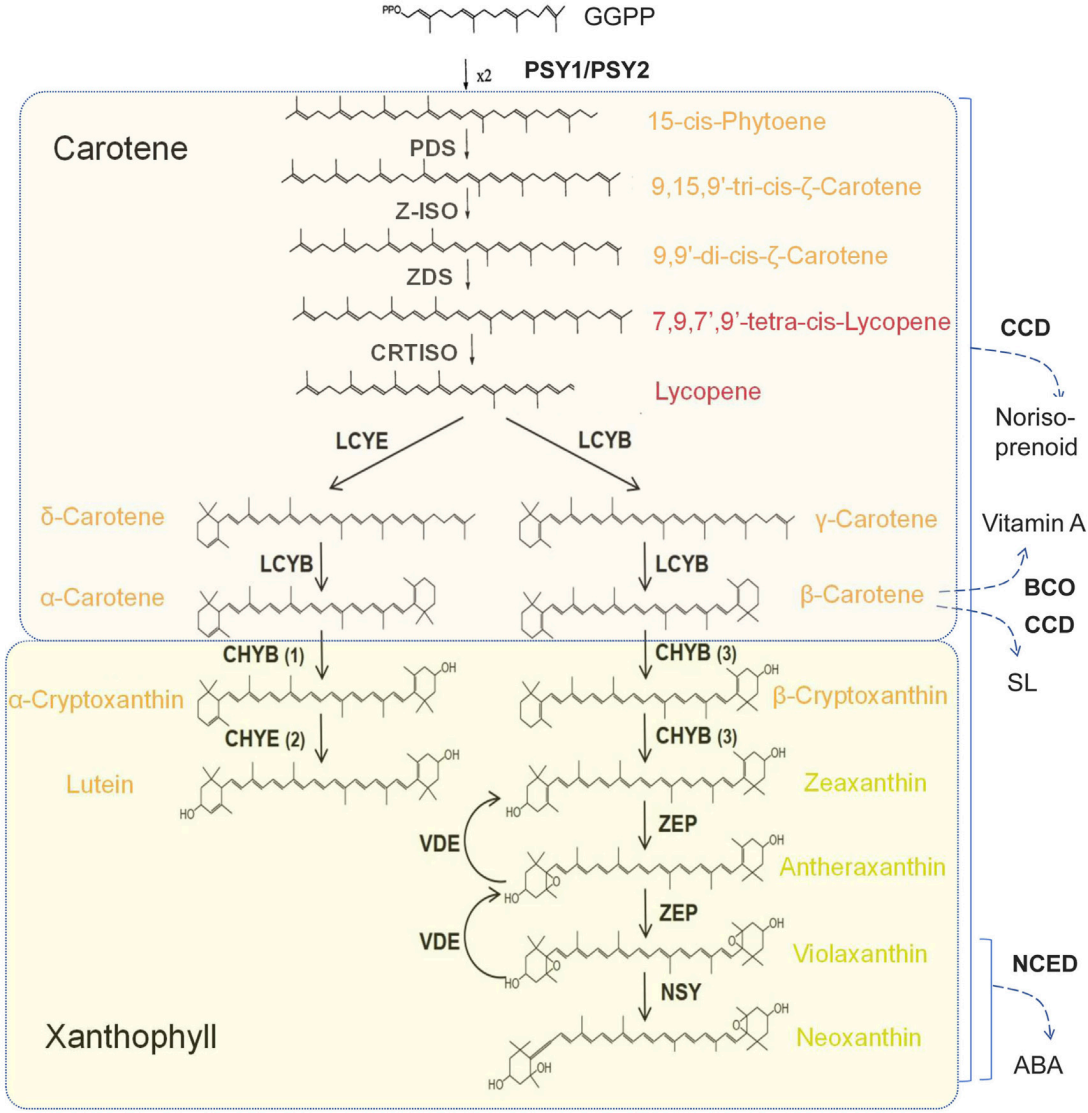
Carotenoids synthesis pathways in plants. PSY, Phytoene synthase; PDS, Phytoenedesaturase; Z-ISO, ε-Carotene isomerase; ZDS, ε-Carotenedesaturase; LCYE, Lycopene δ-cyclase; LCYB, Lycopene β-cyclase; CHYB, β-Carotene hydroxylase; CHYE, ε-Carotene hydroxylase; ZEP, Zeaxanthin epoxidase; VDE, Violaxanthin depoxidase; NSY, Neoxanthin synthase; CCD, Carotenoid cleavage dioxygenases; BCO, β-carotene-15,15′-oxygenase; NCED, Nine-cis epoxycarotenoid dioxygenase.

Carotenoids biosynthesis in plants occurs via the isoprene pathway. Isopentenyl pyrophosphate (IPPC5) serves as the fundamental five-carbon building block for the biosynthesis of carotenoids, which is synthesized via the methylerythritol phosphate pathway (MEP) within plastid. Geranylgeranyl diphosphate (GGPP) is the direct precursor of carotenoid biosynthesis (Walter and Strack, 2011). Under the catalysis of phytoene synthase (PSY), two GGPP molecules condense to form the phytoene which contains 40-carbon atoms (Figure 1). PSY is recognized as one of the key rate-limiting enzymes in the carotenoid synthesis pathway. Subsequently, phytoene undergoes the introduction of four conjugated double bonds through the sequential action of phytoene desaturase (PDS), ε-Carotene isomerase (Z-ISO), ε-carotene desaturase (ZDS), and carotenoid isomerase (CRTISO), ultimately leading to the formation of lycopene (Park et al., 2002; Li et al., 2007).

Lycopene cyclization represents a critical branch point in the carotenoid biosynthesis pathway. There are two lycopene cyclases in plants that can catalyze the formation of double bonds at distinct positions of cyclohexane, resulting in two different products (Zhao et al., 2011). Lycopene β-cyclase (LYCB) catalyzes the conversion of lycopene to γ-carotene, which contains a β-ring, while lycopene ε-cyclase (LYCE) converts lycopene to δ-carotene, containing an ε-ring (Zhao et al., 2011). Subsequently, δ-carotene is further catalyzed by LYCB to yield α-carotene and its derivatives, which possess one ε-ring and one β-ring. In contrast, γ-carotene is catalyzed by LYCB to form β-carotene and its derivatives, while β-carotene contains two β-rings (Niaz et al., 2023).

Both α-carotene and β-carotene serve as precursors for the biosynthesis of xanthophylls. Lutein is produced by the addition of a hydroxyl group to each of the two violone rings of α-carotene, catalyzed by the joint action of ε-carotene hydroxylase (CHYE) and β-carotene hydroxylase (CHYB) (Tian et al., 2004; Kebede and Rahman, 2014). Similarly, β-carotene can be catalyzed with CHYB to produce zeaxanthin by adding a hydroxyl group to each β- rings (Zhang et al., 2022). Subsequently, zeaxanthin is converted into antheraxanthin, a single-ended epoxide, and violaxanthin, a double-ended epoxide, under the action of zeaxanthin epoxidase (ZEP) (Welsch et al., 2018; Niaz et al., 2023). Conversely, violaxanthin can be converted back to antheraxanthin and then to zeaxanthin by the catalytic action of violaxanthin de-poxidase (VDE), which has opposing functionality to ZEP (Walter and Strack, 2011; Jahns and Holzwarth, 2012). Furthermore, the epoxide ring at one end of violaxanthin is cleaved and transformed into neoxanthin by the catalysis of neoxanthin synthase (NSY/NSX) (Neuman et al., 2014). In addition, carotenoids undergo degradation by dioxygenase carotenoid cleavage dioxygenase (CCD) or 9-cis-epoxycarotenoid dioxygenase (NCED), and enter into the biosynthesis pathways leading to the production of abscisic acid (ABA), vitamin A, or strigolactone (SL) (Maoka, 2020).

The expression of genes related to carotenoid synthesis and degradation metabolism plays a pivotal role in determining petal color formation in certain plants (Tian et al., 2003; Zhu et al., 2010). For instance, in the case of lily, the yellow hue of petals is tightly linked to the abundance of gene expression for enzymes such as *PSY*, *PDS*, *ZDS*, *CRTISO* and other genes (Yamagishi et al., 2010). High expression of *CmCCD4a* in white chrysanthemum (*Chrysanthemum morifolium* Ramat.) leads to rapid degradation of carotenoids, thereby resulting in the exhibition of white petals (Ohmiya et al., 2006). Conversely, the mutation of the *CmCCD4a* in yellow chrysanthemum causes the accumulation of carotenoids in petals, resulting in yellow flowers (Yoshioka et al., 2012). Chromoplast is the main organelle for the biosynthesis and storage of carotenoids in petals (Li and Yuan, 2013). Within the chromoplast, carotenoids accumulate within plastoglobulus (PGs), which are specialized lipoprotein particles (Ytterberg et al., 2006). Notably, lutein accumulation is primarily in the form of esters with fatty acids, rather than in its free (non-esterified) form (HorneroMéndez and Mínguez-Mosquera, 2000). Mutations in genes involved in carotenoid esterification would affect the development of PGs and the accumulation of pigments in PGs (Ariizumi et al., 2014), suggesting that carotenoid esterification is also extremely important for the formation of flower colors.

### 2.2 Flavonoids biosynthetic pathways and key genes

**FIGURE 2:**
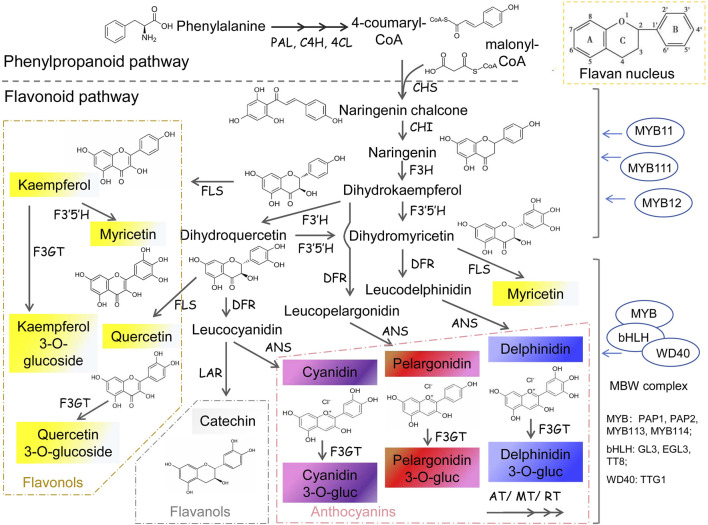
Flavonoids synthesis pathway in plants PAL, phenylalanine ammonialyase; C4H, cinnamate 4-hydroxylase; 4CL, 4-coumaric acid CoA ligase; CHS, chalcone synthase; CHI, chalcone isomerase; F3H, flavanone 3-hydroxylase; F3′5′H, flavonoid 3′ 5′-hydroxylase; F3′H, flavonoid3′-hydroxylase; FLS, flavonol synthase; DFR, Dihydro flavonol reductase; LAR, leucoanthocyanidin reductase; ANS, Anthocyanidin Synthase; F3GT, flavonoid 3-Glucosyl transferase; AT, Acyltransferase; MT, Methyl transferase; RT, Rhamnosyl transferase.

Flavonoids are diverse water-soluble pigments which control the orange, red, purple, and blue colors in petals of most plants. As important secondary metabolites in plants, flavonoids are involved in various growth and development processes. The basic structure of flavonoids is C6-C3-C6 rings (Figure 2), two benzene rings with a phenol hydroxyl group are connected to each other by a central three carbon atom (He and Giusti, 2010). The anabolic pathway for flavonoids begins with the phenylpropanoid pathway, which produces phenylalanine as a precursor. Phenylalanine is then converted into cinnamic acid by the enzyme phenylalanine ammonia-lyase (PAL). Subsequently, cinnamic acid is hydroxylated to form coumaric acid, which enters the flavonoid biosynthetic pathway (Appelhagen et al., 2014; Li, 2014; Shen et al., 2022). Flavonoids encompass a diverse array of compounds, including flavones, ehaleones, flavonols, anthocyanidins, proanthocyanidins, flavanones and isoflavones. Among them, anthocyanins are mainly related to flower color, and the corresponding flavonoid metabolic pathway is called anthocyanin pathway (Harborne, 1986; Saito et al., 2013).

The anthocyanin pathway is highly specialized and involves specific genes encoding enzymes that catalyze key steps in the biosynthetic process. The resulting anthocyanins can further be categorized based on the number of hydroxyl groups present in their B ring. For instance, pelargonidins, which carry a single hydroxyl group, typically impart pink, bright red, or orange hues. Cyanidins, with two hydroxyl groups, predominantly yield purple or red colors. Delphinidins, containing three hydroxyl groups, are responsible for the predominantly blue hues observed in petals (Figure 2).

The regulation of these biosynthetic genes is crucial for controlling the production and accumulation of anthocyanins, thereby influencing the final color phenotype of flowers. Mutations or alterations in these genes can lead to significant changes in petal color, providing valuable insights into the genetic control of flavonoid biosynthesis and its impact on plant phenotype.

From naringenin, various subclasses of flavonoids can be produced through a series of enzymatic reactions catalyzed by specific genes. Multiple genes regulate flavonoid synthesis and their mutations can lead to alterations in flavonoid type and content (Appelhagen et al., 2014). Nowadays, the flavonoid synthesis pathway is well-characterized, and its regulation is not only determined by enzyme-encoding genes but also modulated by various transcription factors, either alone or as part of MBW complexes (Shen et al., 2022). For example, flavonol synthase (FLS) catalyzes the conversion of naringenin into flavonols, while dihydroflavonol 4-reductase (DFR) and anthocyanidin synthase (ANS) are involved in the biosynthesis of anthocyanins. Other key genes involved in flavonoid biosynthesis include flavonoid 3′-hydroxylase (F3H), and flavonoid 3′,5′-hydroxylase (F3′5′H), which modify the structure of flavonoids by introducing hydroxyl groups or methyl groups. For instance, roses, carnations, and chrysanthemums lack the F3′5′H gene, preventing the synthesis of delphinium pigment and resulting in the absence of natural blue petal resources in these plants (Dai and Hong, 2016).

The stability of anthocyanin is closely related to petal color duration. Transferase coding enzymes including GT (Glucosyl transferase), AT (Acyl transferase), MT (Methyl transferase), and RT (Rhamnosyl transferase) catalyze the modifications of anthocyanins in different ways, thereby enhancing their stability within cells (Bombarely et al., 2016; Zhao et al., 2021). And the different positions of acylation and methylation of anthocyanins will also affect the flower color. Subsequently, the synthesized anthocyanins will be transported to the vacuole, a process facilitated by the collaborative action of GST (Glutathione S-transferase, TT19), MATE (Multidrug andtoxic compound extrusion, TT12), and ABC (ATP-binding cassette transporter) transporters (Marinova et al., 2007; Sun et al., 2012; Behrens et al., 2019). Consequently, both the pH level of the vacuole and the presence of metal ions or other types of flavonoids within it impact the coloration of anthocyanins (Hondo et al., 1992).

## 3 Formation and regulation mechanisms of flower color in *Brassica* crops

*Brassica* crops exhibit flower color diversity. The predominant flower color of *Brassica* crops is yellow, although mutant and interspecific hybridization has led to the emergence of distinct petal colors (Chen et al., 1988; Zhang et al., 2000). To date, the reported color variations can mainly be divided into two groups, the yellow series color including white, milky white, milky yellow, yellow, golden yellow, lemon yellow, orange, and yellow-white chimera, and the red series color like orange-red, pink, apricot, red and purple, et al. Recently, significant progress has been made in elucidating the molecular mechanism of flower color formation and regulation in *Brassica* crops.

### 3.1 The inheritance of flower color in *Brassica* crops

The flower color inheritance patterns vary with species even individual plants (Supplementary Table S1). In *Brassica rapa*, yellow flower is dominant, it is reported that compared with yellow flowers, the white, milky white, light yellow and orange flowers are recessive and controlled by 1-2 pairs of genes (Kebede and Rahman, 2014; Zhang et al., 2019; Zhang et al., 2020; Guan et al., 2023). While in *B. oleracea*, the yellow flower is the recessive one, white flower is controlled by a dominant single gene (Han et al., 2019). As for *B. juncea*, the white, milky and light-yellow flowers were controlled by two pairs of recessive genes (Rawat and Anand, 1986; Zhang et al., 2018a). The white flower of *B. carinata* is not completely dominant compared with yellow flower, while the milky white and light-yellow flower are recessive and are controlled by a single pair of genes (Jambhulkar and Raut, 1955; Getinet et al., 1993; Tian, 2011).

*Brassica napus* is one of the most important oil crops and also planted as vegetable, and fodder crops worldwide. The inheritance of flower color in *B. napus* exhibits considerable complexity. Comparative studies have demonstrated that, compared to yellow flower, the white flowers are dominant or incompletely dominant, and governed by one or two loci (Huang et al., 2014; Zhang et al., 2015; Huang et al., 2017). Milky white is recessive relative to milky yellow or yellow, golden yellow and orange flower is recessive relative to yellow flower, light yellow is incomplete dominant relative to lemon yellow, these traits are all controlled by two pairs of genes (Zhang et al., 2000; Liu et al., 2020; Jia et al., 2021). Yellow-white chimeric materials are presumed to be controlled by locally expressed recessive albino genes and exhibit simple recessive traits independent of yellow inheritance or single gene maternal effects (Yu et al., 2004; Yin and Guan, 2013). The studies of red series color flowers primarily focus on *B. napus*. Compared to yellow flower, several studies suggest that the orange and red flower are controlled by two pairs of recessive genes (Dan, 2016; Yao et al., 2017), while other investigations indicate that the orange-red, apricot, and red colors are governed by a single dominant gene (Guo et al., 2021; Ye et al., 2022; Chen et al., 2023). In addition, the red flower trait is recessive when compared with the yellow flower, and was found to be gradually altered in F_2_ population, indicating that the red flower was a quantitative trait of *B. napus* (Jiang, 2021).

Collectively, these findings suggest that the molecular mechanisms underlying single flower color in *Brassica* crops are influenced by multiple genetic pathways. Consequently, further research is required to elucidate the genetic mechanisms that underlie the diverse color phenotypes observed in these *Brassica* crops. What’s more, by analyzing the inheritance pattern of yellow and white flowers of *Brassica* crops, it is found that in species sharing the A genome, such as *B. rapa* and *B. juncea*, the white flower trait is governed by one or two recessive loci, and the yellow flower phenotype is dominant over the white flower (Rahman, 2001; Zhang et al., 2019; Yang et al., 2021; Guan et al., 2023). Conversely, in species harboring the C genome, including *B. oleracea*, *B. napus* and *B. carinata*, the white flower is dominant over yellow flower, and is controlled by one or two nuclear genes (Pearson, 1929; Liu et al., 2004; Huang et al., 2014; Zhang et al., 2015; Han et al., 2019). These observations suggest a strong correlation between flower color inheritance in *Brassica* crops and the genetic relationships among the A, B, and C genomes. However, there are relatively few genetic studies on other flower colors, it needs more results to verify.

### 3.2 Genetic regulation of yellow series flowers in *Brassica* crops

**FIGURE 3:**
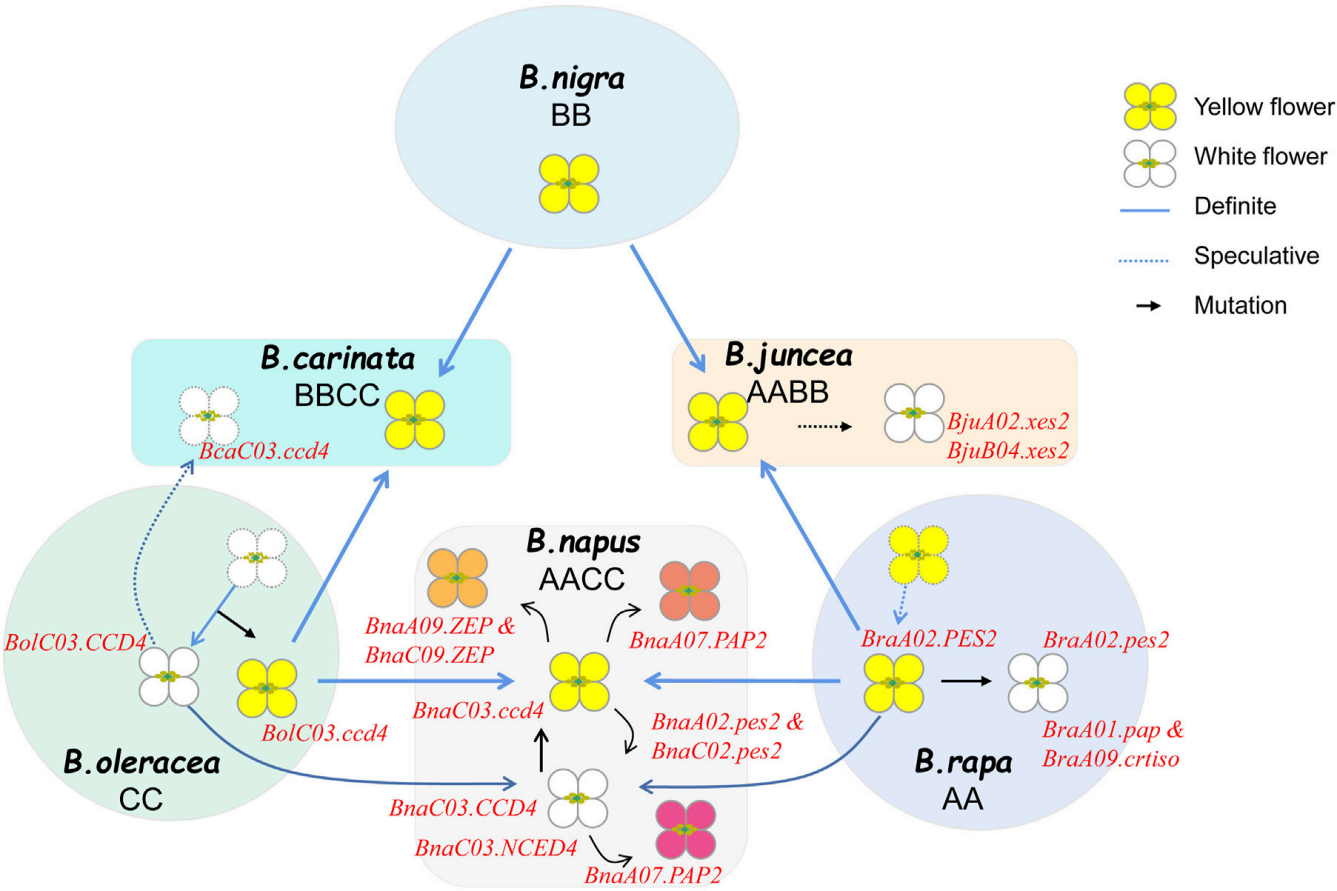
Schematic diagram of flower color formation of the *Brassica* species in the U triangle.

The predominant flower color in *Brassica* crops is yellow, while the most common color variations are from white to orange due to the decrease or accumulation of yellow pigments. To date, several genes that govern the development of yellow series flowers have been identified (Figure 3). In *B. rapa*, two recessive genes, *BrWf1 and BrWf2* that jointly control the formation of white flower, encode a plastid-lipid associated protein (PAP) and a carotenoid isomerase (CRTISO), respectively (Zhang et al., 2019). It is reported that the mutation of *BrA01. PAP (Bra013602)* decrease the accumulation of carotenoid by blocking PG formation and the mutation of *BrA09. CRTISO* (*Bra031539*) decrease the flux of carotenoid biosynthesis pathway, when two genes mutant together, the flower color appeared white. The single nuclear gene *BrWF3* and *BraFC* that controls the white flower trait were found to be the same gene, *Bra032957*, a homolog to *Arabidopsis PES2* involved in fatty acid phytyl-ester synthesis in chloroplasts. A premature stop codon mutation within *Bra032957* disrupted the gene function, leading to increase in abnormal chromoplasts with irregularly structured PG and decrease in xanthophylls in the white petals of flowers (Yang et al., 2021; Guan et al., 2023). Knockout mutants of the two orthologous genes (*BnaA02. PES2-2 and BnaC02. PES2-2*) of *BraFC* were constructed in *B. napus*, and showed white petals (Guan et al., 2023). In *B. juncea*, two independent recessive genes, *Bjpc1*(*BjuA02. xes2*) and *Bjpc2 (BjuB04. xes2, BjuB027334)*, controlling the white flower trait, are also homologous to *AtPES2* (Zhang et al., 2018a; Zhang et al., 2018b; Li et al., 2023), which mean that the carotenoid esterification contribute a lot in the formation of white flowers in *Brassica* crops.

Carotenoid lytic dioxygenase genes also play important roles in the formation of white flowers. Until now, In *B. oleracea* and *B. napus*, all reported white flower loci were mapped to the chromosome C03, and *CCD4*, a carotenoid cleavage dioxygenase 4 gene was confirmed to be the single nuclear gene controlling the white flower formation (Zhang et al., 2015; Han et al., 2019; Yang et al., 2019). Zhang et al. (2015) reported that *BnaC03. CCD4 (BnaC03G0619000NO)* cleaves δ-carotene and/or α-carotene into volatile α-ionone, which partitions most of the flux in the carotenoid biosynthesis pathway and results in white flowers with decreased yellowish pigments like lutein. And the insertions and deletions occurring in the coding regions of *BnaC03. CCD4* disrupted its function and resulted in a significantly higher accumulation of carotenoids in petals of yellow *B. oleracea*, *B. napus* and *B. carinata*, which became the prevalent flower color across these species (Zhang et al., 2015). Jia et al. (2021) also confirmed that *BnaC03. NCED4* (*BnaC03G0710000ZS, a homologous gene of BnaC03. CCD4*) can turn rape petals from yellow to white by degrading the carotenoids content. In *B. oleracea*, the results of gene localization and functional analysis showed that *BolC.cpc-1*(*Bol029878*) or *BoCCD4 (Bol029878)* is the candidate gene for white flower formation, which is highly expressed in white cabbage, but extremely low in yellow flower, and when mutant, the white flowers turn yellow (Han et al., 2019; Xu et al., 2019; Yang et al., 2019).

The reported novel flower colors of *Brassica* crops primarily encompass gold-yellow, and orange hues. It has been observed that as the petal color transitions from white to yellow and then to golden yellow, the total carotenoid content increases (Zeng, 2013). Notably, the content of lycopene exhibits the most significant difference between golden yellow with other colors. This differential lycopene content may be attributed to the highest expression of *BnPSY* and relatively low expression of *BnLYCE*, leading to a significantly higher synthesis of lycopene in golden yellow flowers compared to other colors (Zeng, 2013). *BnaA08. PDS3* encodes phytoene desaturase 3, one of the rate-limiting enzymes in carotenoid biosynthesis pathways, and the mutation of *BnaA08. PDS3 (BnaA08g17170D)* results in decreased carotenoids and causes yellowish-white petals in *Brassica* napus (Zhao et al., 2021). The genetic loci associated with orange flower color have been primarily mapped on chromosome A09 and C09 (Yao et al., 2017; Zhang et al., 2019; Ding et al., 2019; Zhang, 2021). Specifically, *BnaA09. ZEP* (*BnaA09g07610D*) and *BnaC09. ZEP* (*BnaC09g075550D*) have been identified as the candidate genes that control the orange flower trait, as these genes were typically expressed in yellow petals but not in orange petals (Liu et al., 2020; Zhang, 2021). Functionally, these two genes exhibit redundancy in carotenoid synthesis, facilitating the restoration of orange flower color to yellow (Liu et al., 2020). RNA-sequencing studies have further revealed that β-carotene is the primary contributor to the formation of orange flowers. Decreased expression levels of *CHYB* and *ZEP* in orange petals lead to an accumulation of β-carotene (Zhang et al., 2019). This accumulation of β-carotene, in conjunction with the genetic regulation of *ZEP*, contributes to the distinct orange hue observed in *Brassica* crops. In summary, carotenoids play a pivotal role in determining flower coloration in *Brassica* crops, and of these pigments are intricately involved in the transition among yellow series color.

### 3.3 Genetic regulation of red series flower in *Brassica* crops

As is well-established, anthocyanins significantly influence the accumulation of red pigments in plants. In *Brassica* crops, *BnaA07. PAP2* (*BnaA07G0287000ZS*) has been identified as a key regulator of the orange-red, apricot and red color in *B. napus* flowers (Jia et al., 2021; Ye et al., 2022; Chen et al., 2023). This gene modulates anthocyanin biosynthesis by inducing the expression of *Bna. DFR* and *Bna. ANS*, and the combined accumulation of anthocyanins and carotenoids in petals leads to a transition of flower color from orange to red. Knockout of *BnaA07. PAP2* disrupts anthocyanin synthesis, resulting in a shift in petal color from orange-red to yellow (Jia et al., 2021). Besides, it has been reported that the key genes of anthocyanin biosynthesis, *ANS*, *DFR*, and *UF3GT*, are significantly more highly expressed in red and apricot petals compared to white and yellow petals. The RNA interference of *BnaA03. ANS* would alter petal colors from raspberry red to beige red and zinc yellow under different interference levels (Hao et al., 2022). The expression of *BnaA10.F3′H* (*BnaA10g23330D*) affects the synthesis of downstream peonidin and delphinidin and is a key gene regulating the purple color of petals in *B. napus* (Li et al., 2023). Despite these advancements, the precise molecular mechanism underlying the formation of red flowers remains incompletely understood. Future studies are needed to further elucidate the complex interactions between anthocyanins and other genetic and environmental factors that govern flower coloration in *Brassica* crops.

### 3.4 Carotenoids and anthocyanins contribute to the flower color diversity of *Brassica* crops

It is confirmed that carotenoids are the key pigments for yellow series flowers, whereas anthocyanins promote the formation of red series flower in *Brassica* crops. Manipulating the endogenous pigment levels within cells can facilitate transitions in petal color, ranging from white to yellow or red. The total carotenoids content is a culmination of biosynthesis, degradation and stable storage processes (Yang et al., 2019). While several factors involved in these processes have been verified as regulators of the petal color of *Brassica* crops, the intricate molecular mechanisms underlying the flower color formation through the flavonoids biosynthesis pathway in *Brassica* crops remain largely enigmatic.

Transcription factors (TFs) play a pivotal role in orchestrating the expression of structural genes involved in carotenoid and flavonoid biosynthesis, thus influencing flower color (Hopkins and Rausher, 2011). MYB, WD40, and bHLH are three key transcription factor families that has reported to significantly affected petal color formation (Ramsay and Glover, 2005). Notably, an R2R3-MYB transcription factor encoding gene, *BnaA07. PAP2* was cloned through map-based cloning strategy, and the introduction of *BnaA07. PAP2* ^*In-184-317*^ into yellow-flowered *B. napus* triggered a widespread transcriptional activation of the anthocyanin biosynthetic pathway, ultimately resulted in apricot flowers (Ye et al., 2022). Moreover, two additional genes, a MYB (*BnaMYBL2*) and a bHLH (*BnaTT8*) gene, exhibited similar expression pattern of *BnaA07. PAP2* during flower developments in different-colored petals. This consistency underscores the importance of MYB and bHLH in regulating anthocyanin-based color formation in *Brassica* (Hao et al., 2022). The carotenoid biosynthetic pathway is regulated primarily by a diverse array of TF families, including R2R3-MYB, MADS-box, NAC, bHLH, SBP-box, AP2/ERF, HD-ZIP, NF-Y, and WRKY (Stanley and Yuan, 2019). Utilizing multi-omics approach, amounts of transcription factors involved in regulating carotenoid metabolism have been identified which showed dramatical differential expression patterns between white-flowered and yellow-flowered petals across all development stages (Jia et al., 2021; Zhang et al., 2022). Among these genes, six TFs, including BnMYB106 (BnaCnng29120D), BnMADS (BnaC02g00490D), BnHD-ZIP (BnaA09g18250D, BnaCnng02160D), BnNFYA1 (BnaA03g04040D), and BnWRKY22 (BnaCnng02000D), showed expression patterns that align with white flower gene *BnNCED4* (Jia et al., 2021). Similarly, the expressive pattern of two key TF-encoding genes, *Bo2g151880* (WRKY) and *Bo3g024180* (SBP) were consistent with the BoCCD4 (Zhang et al., 2022). This suggests that these TFs may interact with *CCD4* to jointly regulate carotenoid biosynthesis in *B. napus* and *B. oleracea* respectively, but the interaction between them remain to be explored. Collectively, these studies provides new insights into the molecular regulatory mechanism of carotenoids and flavonoids underlying petal color formation in *Brassica* crops.

Notably, recent studies also revealed an intriguing interaction between carotenoids and anthocyanins, resulting in the production of red flowers in *B. napus* (Liu et al., 2020; Hao et al., 2022). Previous studies reported that most of the epicatechin, quercetin, and isorhamnetin derivates were found in red and pink petals of *B. napus*, while kaempferol derivates were in yellow and pale white petals (Yin et al., 2019). And the metabolome analysis has further elucidated that apricot coloration resulted from the accumulation of yellow lutein and red anthocyanins, whereas pink color arises from the combined presence of colorless carotene and red anthocyanins (Ye et al., 2022). These findings suggest that the interplay between the carotenoids and flavonoids biosynthesis pathways significantly impacts flower color formation in *Brassica* crops. Thus, manipulating the abundance ratio of key carotenoids and flavonoids, including anthocyanins, through the regulation of involved genes or pH-regulating genes, may serve as a promising strategy for the creation of novel flower colors in these crops.

## 4 The genetic mechanisms underlying the evolution of yellow and white flower colors in *Brassica* crops

As is well known, the A, B and C genomes of *Brassica* are closely related, exhibiting high synteny within groups, particularly between A and C genomes (Prakash et al., 2009). In species sharing the A genome, the yellow flower phenotype is typically dominant over the white flower. Conversely, in species harbor the C genome, the white flower prevails. The similarity in genetic pattern of flower colors among *Brassica* crops may suggests a conserved evolution of key regulatory genes. For instance, the white-flower controlling gene *CCD4* has been cloned on chromosome C03 of *B. oleracea, B. napus* and *B. carinata*, the fatty acid phytyl-ester function of *A02. PES2* is conserved in *B. rapa*, *B. napus* and *B. juncea*, and their homologous genes on B or C genome are functional, jointly contributing to the white flower formation. These observations point to the varying genetic mechanisms underlying flower color evolution in *Brassica* crops as being closely tied to the evolutionary history of these species. Further studies into the genetic mechanisms driving these patterns could provide valuable insights into the molecular basis of flower color evolution in *Brassica* and potentially other plant species.

*B. napus* as an allopolyploid originated from the hybridization of *B. rapa* with *B. oleracea* exhibits a more complex genetic basis for flower colors. The early reported petal color mutations including natural or artificial synthesized in *B. napus* is white, which was supposed to be transferred from white cabbages (Heyn, 1977; Chen et al., 1988; Qi and Fu, 1992). Zhang et al. (2002) made the crosses between the yellow-petalled *B. rapa* with white-petalled *B. oleracea* and cream–yellow-petalled *B. oleracea* to produce resynthesized *B. napus* (2n = 38, AACC or CCAA) and sesquidiploids (2n = 29, AAC or CAA). The petal color parameters of different genome compositions revealed that the C-genome white petal gene was partially epistatic over the A-genome yellow petal gene. The degree of white color increases with an increased dosage of C-genome and with the presence of the *B. oleracea* cytoplasm. Currently, the formation of yellow and white flower of *B. napus* is one of the most extensively studied topics, and several key genes have been cloned from both A and C genomes. Consequently, the evolutionary relationship between yellow and white flower colors in *B. rapa*, *B. oleracea*, and *B. napus* has been established. For instance, Zhang et al. (2015) cloned the white flower gene *BnaC3. CCD4* and identified four variations (M1-M4) in *Brassica* crops, which destroyed the gene function resulting in yellow-flowered lines. All of the four mutation types presented in *B. oleracea* with yellow flowers, but only M1 type existed in *B. napus*, and only M1 type and M4 type existed in *B. carinata*. This might suggest that the white flower gene mutated originally in *B. oleracea* and was subsequently preserved in interspecific hybrids, leading to yellow flowers of *B. oleracea, B. napus* and *B. carinata*, which has been confirmed in the research of Han et al. (2019). Collectively, in consideration of the inheritance patterns of yellow and white flowers of *Brassica* crops, it is likely that the yellow *B. rapa* and white *B. oleracea* might be the ancestral type, and in the origin center of *B. napus*, the yellow *B. rapa* crossed with the yellow-flowered *B. oleracea*, hence the yellow *B. napus* formed. In summary, the significance of considering genetic relationships and evolutionary history becomes evident when examining phenotypic traits in *Brassica* crops. Future research should further explore the genetic mechanisms controlling flower color and their impact on crop evolution and domestication.

## 5 Innovation and multiple utilization of *Brassica* crops with colorful flowers

The variations in flower color among *Brassica* crops primarily arise from natural variation, artificial mutagenesis, interspecific hybridization, and genetically modified (Li et al., 2022). Among these methods, interspecific hybridization is the most commonly employed for enhancing crop characteristics, and the target flower colors could be transferred into other *Brassica* crops (Li et al., 2023). For example, there are no red petals in natural variation of rapeseed but radish does. The rape accessions with orange-red flowers were obtained from the high generation backcross progenies of *B. napus* and the wide hybrids between *B. napus* and *Raphanus sativus* L. (Chen et al., 2023). However, it is noted that wide hybridization can also result in the unintentional transfer of undesirable traits, attributed to linkage drag. To address this, transgenics and gene editing techniques can be employed to achieve directional improvement in flower color. White flowers have been innovated by knocking out two homologous of *BraA02.PES2-2* in the yellow-flowered *B. napus* with the CRISPR/Cas9 system (Guan et al., 2023). Furthermore, the introduction of specific metabolic enzymes, controlled by a flower-specific promoter, can create novel color phenotypes without compromising other traits, as demonstrated by Zhu et al. (2010). For instance, the ectopic overexpressing of *OvPAP2* gene driven by the petal-specific promoter XY355 leads to red anthers and red petals in *B. napus* (Fu et al., 2018).

Novel flower colors in *Brassica* crops play a pivotal role in enhancing their popularization and application. Flower color serves as a key morphological marker in *Brassica* crops. Due to the color variations on leaves, stems and petals, *Brassica* crops have been used as ornamental plants in gardens and landscapes, as well as cut flowers in pots and containers. The increasing demand for visually appealing plants underscores the need to cultivate more *Brassica* crops with colorful flowers. Moreover, *Brassica* crops, particularly those with colorful petals, are rich in nutrients and bioactive compounds, including carotenoids, flavonoids, vitamins, minerals, and antioxidants, et al. These compounds make them a valuable source of natural nutrients and health-promoting compounds in functional foods, dietary supplements, and nutraceutical products (Zayed et al., 2022). Besides, these crops may also exhibit resistance to environmental stress (Verma et al., 2023). Studies have shown that certain *Brassica* species can accumulate heavy metals and other contaminants from soil and water (Zhang et al., 2023). Planting these crops in contaminated areas could provide a sustainable and cost-effective method for removing harmful substances from the environment. The unique aesthetic appeal, nutritional content, and environmental remediation potential of *Brassica* crops with colorful petals highlight their significance in modern horticulture and agriculture.

## 6 Conclusion and perspective

Flower color, a key morphological trait, is governed by multiple loci with complex genetic basis in *Brassica* crops. We outline the recent progress of different flower colors formation and regulation of six representative *Brassica* crops. These crops have a closely genetic relationship, which can help to elucidate the genetic mechanisms of flower color formation and evolution. It is found that carotenoids are the basic pigments which control the common colors of *Brassica* crops, yellow and white series color of petals, and anthocyanins promote the formation of red series flowers. Based on the cloned key genes involving in the carotenoids and anthocyanins anabolic pathways, it is possible to create more colorful resources with biotechnological methods and approaches, such as hybridization, transgenics and gene editing. Thereby, it can further increase the application value and economic value of *Brassica* crops. To gain deeper understanding of flower color evolution, we delve into the genetic relationship among *B. rapa*, *B. oleracea* and *B. napus*. By analyzing the inheritance patterns within the A and C genomes, we postulate that the yellow-flowered *B. rapa* and white-flowered *B. oleracea* might be the ancestral types. By the way, there are relatively few studies on the flower color formation and evolution of *B. nigra* and *B. carinata* that sharing the B genome.

Flower color, easily observable without specialized instrumentation or reagents, serves as a convenient morphological marker with diverse applications in plant breeding. It is frequently used to assess the natural outcross rate of varieties, verify the purity of parents and hybrids in heterosis utilization systems, and as a linkage marker for targeted breeding traits (Yin and Guan, 2013). A successful selection and breeding of an excellent restorer line with orange flowers, facilitated by the close linkage of molecular markers to the orange flower color genes *Bnpc1* and *Bnpc2*, has demonstrated its utility. This novel flower color enables seed purity reach to 98% during seed production (Zhang, 2021). Given the limited number of flower color-controlling genes reported, it would be highly beneficial to integrate these genes with other superior agronomic trait-related genes during breeding screening in the future. These strategies hold promise for the development of *Brassica* crops with enhanced performance and reduced environmental risk. In conclusion, the exploration of flower color genetics and its application in *Brassica* crops offers promising avenues for future research, with potential to enhance breeding efficiency, economic value, and crop resilience.
