# Supplementary material for: The formation and evolution of flower coloration in Brassica crops
Source: Front Genet. 2024 May 30;15:1396875. doi: 10.3389/fgene.2024.1396875 (PMC11177764; doi:10.3389/fgene.2024.1396875)
Supplement: Supplementary file 1 [file Table1.DOCX]

**Supplemental Table S1 The inheritance of flower color in Brassica crops**

| Species | Flower Colors | Dominant or Recessive | Inheritance (Loci) | References |
| --- | --- | --- | --- | --- |
| *B.rapa* | White | Recessive | 1 | Mohammad et al., 1942 |
|  | Milky Yellow | Recessive | 2 | Alam and Aziz, 1954 |
|  | Milky White | Recessive | 1 | Cours, 1977 |
|  | Orange | Recessive | 1 | Cours, 1977 |
|  | Milky White | Recessive | 1 | James et al., 1980 |
|  | Milky White | Recessive | 1 | Orakwue and Crowder, 1983 |
|  | Milky Yellow | Recessive | 1 | Séguin-Swartz, 1988 |
|  | White | Recessive | 1 | Zhang and Li, 1999 |
|  | White | Recessive | 1 | Singh et al., 2014 |
|  | Creamy White | Recessive | 1 | Kebede and Rahman, 2014 |
|  | Orange | Recessive | 1 | Zhang, 2019 |
|  | White | Recessive | 2 | Zhang et al., 2020 |
|  | White | Recessive | 1 | Tian, 2021 |
|  | White | Recessive | 1 | Yang et al., 2021 |
|  | White | Recessive | 1 | Guan et al., 2023 |
| *B. nigra* | / | / | / | / |
| *B. oleracea* | White | Dominant | 1 | Pearson, 1929 |
|  | Milky White | Recessive | 1 | Anstey and Moore, 1954 |
|  | Milky White | Recessive | 1 | Anestey et al., 1954 |
|  | Milky White | Recessive | 1 | Sampson, 1966 |
|  | White | Dominant | 1 | Snogerup et al., 1990 |
|  | White | Dominant | 1 | Kianian and Quiros, 1992 |
|  | White | Dominant | 1 | Han, 2019 |
|  | White | Dominant | 1 | Yang et al., 2019 |
| *B. juncea* | Milky White | Recessive | 2 | Sun, 1945 |
|  | Milky Yellow | Recessive | 2 | Alam and Aziz, 1954 |
|  | White | Recessive | / | Anand and Mishra, 1985 |
|  | White | Recessive | 2 | Chen and Tong, 1985 |
|  | Milky White | Recessive | 2 | Bhuiyan, 1986 |
|  | Milky White | Recessive | 2 | Rawat and Anand, 1986 |
|  | White | Recessive | 2 | Zhang et al., 2018 |
| *B. napus* | Milky Yellow | Incompletely Dominant | 2 | Sylvén, 1927 |
|  | White | Dominant | 2 | Heyn, 1977 |
|  | Milky White | Recessive | / | Heyn, 1977 |
|  | White | Dominant | 1 | Quazi, 1988 |
|  | White | Dominant | 1 | Chen et al., 1988 |
|  | White | Dominant | 1 | Chen and Heneen, 1990 |
|  | White | Incompletely Dominant | 1 | Qi and Fu, 1992 |
|  | White | Dominant | 1 | Woods et al., 1997 |
|  | Orange Red | Recessive | 2 | Li et al., 1999 |
|  | Golden Yellow | Recessive | 2 | Zhang et al., 2000 |
|  | White | Incompletely Dominant | 1 | Zhang et al., 2000 |
|  | White | Dominant | 1 | Rahman, 2001 |
|  | White | Dominant | 1 | Liu, 2004 |
|  | White | Incompletely Dominant | 1 | Dong, 2005 |
|  | White | Dominant | 1 | Liu, 2005 |
|  | White | Incompletely Dominant | 2 | Tian, 2007 |
|  | White | Incompletely Dominant | 1 | Chen et al., 2009 |
|  | White | Codominant | 1 | Wen et al., 2010 |
|  | White | Dominant | Quantitative | Tian, 2011 |
|  | White | Incompletely Dominant | 1 | Ban, 2013 |
|  | White | Incompletely Dominant | 1 | Yin and Guan, 2013 |
|  | White | Incompletely Dominant | 1 | Huang et al., 2014 |
|  | White | Dominant | 1 | Zhang et al., 2015 |
|  | Orange | Recessive | 2 | Dan, 2016 |
|  | White | Dominant | 1 | Huang et al.,2017 |
|  | Orange | Recessive | 2 | Yao, 2017 |
|  | Orange Red | Recessive | 2 | Zhang, 2020 |
|  | Orange | Recessive | 2 | Liu, 2020 |
|  | Orange Red | Dominant | 1 | Guo, 2021 |
|  | Orange Red | Dominant | 1 | Jia, 2021 |
|  | Red | Recessive | Quantitative | Jiang, 2021 |
|  | White | Dominant | 1 | Chen, 2021 |
|  | White | Dominant | 1 | Jia, 2021 |
|  | Yellowish-White | Recessive | 1 | Zhao et al., 2021 |
|  | Apricot | Dominant | 1 | Ye et al., 2022 |
|  | Orange Red | Dominant | 1 | Zhou, 2022 |
|  | Red | Dominant | 1 | Chen et al., 2023 |
| *B. carinata* | White | Incompletely Dominant | 1 | Jambhulkar and Raut, 1955 |
|  | Milky White | Recessive | 1 | Getinet, 1993 |
|  | Milky Yellow | Recessive | 1 | Guo, 2010 |
